# Association between colorectal cancer, the frequency of *Bacteroides fragilis*, and the level of mismatch repair genes expression in the biopsy samples of Iranian patients

**DOI:** 10.1186/s12876-024-03169-z

**Published:** 2024-02-23

**Authors:** Nooshin Nazarinejad, Bahareh Hajikhani, Amir Abbas Vaezi, Farzaneh Firoozeh, Fatemeh Sameni, Somayeh Yaslianifard, Mehdi Goudarzi, Masoud Dadashi

**Affiliations:** 1 Department of Microbiology, School of Medicine, Alborz University of Medical Sciences, Karaj, Iran; 2 Department of Microbiology, School of Medicine, Shahid Beheshti University of Medical Sciences, Tehran, Iran; 3 Department of Internal Medicine, Alborz University of Medical Sciences, Karaj, Iran; 4 Department of Microbiology, Faculty of Medicine, Shahed University, Tehran, Iran; 5 Non-Communicable Diseases Research Center, Alborz University of Medical Sciences, Karaj, Iran

**Keywords:** Colorectal cancer, *mlh1*, *msh2*, *msh6*, *Bacteroides fragilis*

## Abstract

**Background:**

Deficient DNA mismatch repair (MMR) can cause microsatellite instability (MSI) and is more common in colorectal cancer (CRC) patients. Understanding the carcinogenic mechanism of bacteria and their impact on cancer cells is crucial. *Bacteroides fragilis* (*B. fragilis*) has been identified as a potential promoter of tumorigenesis through the alteration of signaling pathways. This study aims to assess the expression levels of *msh2*, *msh6*, *mlh1*, and the relative frequency of *B. fragilis* in biopsy samples from CRC patients.

**Materials and methods:**

Based on the sequence of *mlh1, msh2*, and *msh6* genes, *B. fragilis* specific *16srRNA* and bacterial universal *16srRNA* specific primers were selected, and the expression levels of the target genes were analyzed using the Real-Time PCR method.

**Results:**

Significant increases in the expression levels of *mlh1*, *msh2*, and *msh6* genes were observed in the cancer group. Additionally, the expression of these MMR genes showed a significant elevation in samples positive for *B. fragilis* presence. The relative frequency of *B. fragilis* in the cancer group demonstrated a significant rise compared to the control group.

**Conclusion:**

The findings suggest a potential correlation between the abundance of *B. fragilis* and alterations in the expression of MMR genes. Since these genes can play a role in modifying colon cancer, investigating microbial characteristics and gene expression changes in CRC could offer a viable solution for CRC diagnosis.

## Introduction

Colorectal cancer (CRC) is one of the most prevalent malignancies afflicting both men and women [1]. Globally, CRC ranks as the second cause of cancer-related deaths, claiming the lives of many, and is the third most common cancer worldwide. Fortunately, the incidence of CRC in Iranian individuals is comparatively lower than in Western countries [2]. As of 2020, reports indicate that 1.9 million people are diagnosed with CRC annually [3, 4]. Given its considerable lethality, swift and early diagnosis and intervention become imperative [5]. Individuals with CRC exhibit noticeable alterations in gut microbiota compared to healthy individuals. Notably, there is an increase in the presence of *B. fragilis*, Fusobacterium, Enterobacteriaceae, Campylobacter, Erysipelotrichaceae, Collinsella, and Peptostreptococcus in the faeces of CRC patients. Numerous studies underscore the significance of *B. fragilis* as an enterotoxin-producing bacterium, playing a pivotal role in the initiation and progression of CRC. This involvement occurs through modulation of the mucosal immune response, epithelial cell modification, and the induction of adenoma in the primary stages of CRC [6–9].

Recent investigations confirm the increase of toxigenic *B. fragilis* species in CRC patients. Furthermore, the presence of toxigenic *B. fragilis* markers has been validated in the colon and terminal ileum of patients with ulcerative colitis, a population prone to developing colon cancer [10, 11]. It is demonstrated that *B. fragilis* is a predominant and consistent pathogen in stool mucosa and colon tissue samples of CRC patients [12].

While CRC primary diagnosis conventionally relies on colonoscopy, molecular markers such as carcinoembryonic antigen in serum are employed in clinical settings for CRC diagnosis [13]. However, identifying markers indicative of the transformation from adenomatous polyp to adenocarcinoma in the disease’s early stages remains elusive [14]. Addressing this gap, the identification of diagnostic markers could expedite CRC diagnosis and impede its progression.

The Mismatch Repair (MMR) system, integral to DNA homeostasis, is among the enzyme systems crucial for maintaining genomic stability. MMR loss leads to the rapid accumulation of potential mutations, predisposing individuals to specific cancer types [15].

Mutations in MMR proteins result in Microsatellite Instability (MSI), a genomic instability syndrome implicated in Lynch syndrome and gastrointestinal cancers. Lynch syndrome primarily arises from germ cell mutations, predominantly in mlh1 or msh2, and to a lesser extent in msh6 and rarely pms2 [13]. MMR genes encode proteins that recognize and repair errors that occur during cell replication. In individuals with mutations in MMR genes, such as *msh2*, *msh6*, and *mlh1*, the risk of CRC is significantly increased [14].

Scientific evidence indicates a substantial increase or decrease in the relative expression of MMR system genes in various human cancers [16]. Recent studies underscore a significant elevation in the relative expression of MMR system genes in CRC patients. Consequently, evaluating the extent to which their expression fluctuates in different diseases can serve as a biomarker for cancer diagnosis and treatment, particularly in the context of colorectal cancer. In this study, the expression of *mlh1, msh2, and msh6* genes and the relative presence of *B. fragilis* in biopsy specimens of patients with CRC and healthy individuals were investigated by Real-Time PCR to determine whether the presence of this bacterium affects the expression of selected genes that are involved in CRC development.

## Method

### Sampling

In this study, two separate groups of individuals were evaluated. The first group consisted of twenty healthy individuals suspected of CRC who underwent a colonoscopy, while the second group included a total of 40 patients with CRC. Colonoscopy biopsies were got from the right (from the cecum to transverse colon) and left (from descending colon to the rectum) colons of patients. Tissue biopsies were collected in Transystem tubes containing normal saline and RNA-later, and were kept at − 20 °C until analysis.

### DNA and RNA extraction and cDNA synthesis

Biopsy samples have been extracted using special DNA and RNA extraction kits (ROJE Company- Iran) to analyze tissue samples. In the following step, a spectrophotometer (Nano Drop, 2000) was used to measure the concentration and purity of the extracted DNA. As well, cDNAs were synthesized using a cDNA synthesis kit (RT-Roset, ROJE Company- Iran).

### Real-time PCR

In order to run Real-Time PCR, specific primers mentioned in Table 1 were utilized to assess the selected genes expression modification and the relative abundance of *B. fragilis*. Quantitative PCR reactions were performed on Real-Time PCR Applied Biosystems 7900 using SYBR® select Master Mix in 20 µl reactions. Cycle conditions for the *mlh1, msh2*, and *msh6* genes were as follows: 95 ° C for 10 min and 40 cycles at 95 ° C for 20 s, 55 ° C for 30 s and 72 ° C for 30 s. Cycle conditions for the detection of *B. fragilis* were as follows: 95 ° C for 10 min, and 40 cycles at 95 ° C for 20 s, 56 ° C for 30 s, and 72 ° C for 30 s.

**Table 1:** Primers used in this study

Name	Sequences (5’ − 3’)	Reference
*mlh1*-F	GTGCTGGCAATCAAGGGACCC	(1)
*mlh1*-R	CACGGTTGAGGCATTGGGTAG	
*msh2-*F	CATCCAGGCATGCTTGTGTTGA	(1)
*msh2-*R	GCAGTCCACAATGGACACTTC	
*msh6-*F	TGATGACAGCCCAACAAGGG	(1)
*msh6-*R	AGTTGTGCCTACCTCCATCT	
*16srRNA-*F	TCAGGAAGAAAGCTTGCT	(2)
*16srRNA-*R	CATCCTTTACCGGAATCCT	
*gapdh-*F	ATGTTCGTCATGGGTGTGAA	(3)
*gapdh-*R	ATGTTCGTCATGGGTGTGAA	
*16srRNA-*Universal*-*F	AGMGTTYGATYMTGGCTCAG	(4)
*16srRNA-*Universal*-*R	GCTGCCTCCCGTAGGAGT	

1. Dadashi M, Hajikhani B, Faghihloo E, Owlia P, Yaslianifard S, Goudarzi M, et al. Proliferative effect of FadA recombinant protein from Fusobacterium nucleatum on SW480 colorectal cancer cell line. Infectious Disorders-Drug Targets (Formerly Current Drug Targets-Infectious Disorders). 2021;21(4):623–8

2. Wang I-K, Lai H-C, Yu C-J, Liang C-C, Chang C-T, Kuo H-L, et al. Real-time PCR analysis of the intestinal microbiotas in peritoneal dialysis patients. Applied and environmental microbiology. 2012;78(4):1107–12

3. Wang J, Luo X, Cai S, Sun J, Wang S, Wei X. Blocking HOTAIR protects human chondrocytes against IL-1β-induced cell apoptosis, ECM degradation, inflammatory response and oxidative stress via regulating miR-222-3p/ADAM10 axis. Int Immunopharmacol. 2021;98:107903

4. Dong Z, Chen B, Pan H, Wang D, Liu M, Yang Y, et al. Detection of Microbial 16 S rRNA Gene in the Serum of Patients With Gastric Cancer. Frontiers in Oncology. 2019;9

### Reference gene for qPCR

The *gapdh* cellular gene was applied to normalize the target genes expression in biopsy samples. Also, the bacterial universal *16srRNA* gene was used as a reference gene to investigate the relative abundance of *B. fragilis* (Table 1). In order to ensure the accuracy of the results, all qPCR reactions were conducted in duplicate for controls and tests.

### Statistical analysis

Biopsy samples from the control group (*n* = 20) and cancer group (*n* = 40) in terms of presence, relative frequency of *B. fragilis*, and relative expression of *mlh1*, *msh2*, and *msh6* genes were analyzed. The formula 2^−ΔΔCt^ was used to determine the relative expression of each mentioned MMR genes to *gapdh* RNA.

ΔΔCt = ΔCt (Target)– ΔCt (Reference).

The following formula was used to calculate the fold change in the expression of target genes.

2^−(ct target − ct reference) Tumor^**/** 2^−(ct target − ct reference) normal^

Data analysis was performed using SPSS version 21 and PRISM software version 8. Quantitative data were summarized as mean and reaction progression deviation. Quantitative data were checked for normal distribution, and if normality test was passed, analysis of variance (Non-parametric ANOVA) with a significant level (*P* value < 0.05) was used.

## Results

### Samples

**Fig. 1:**
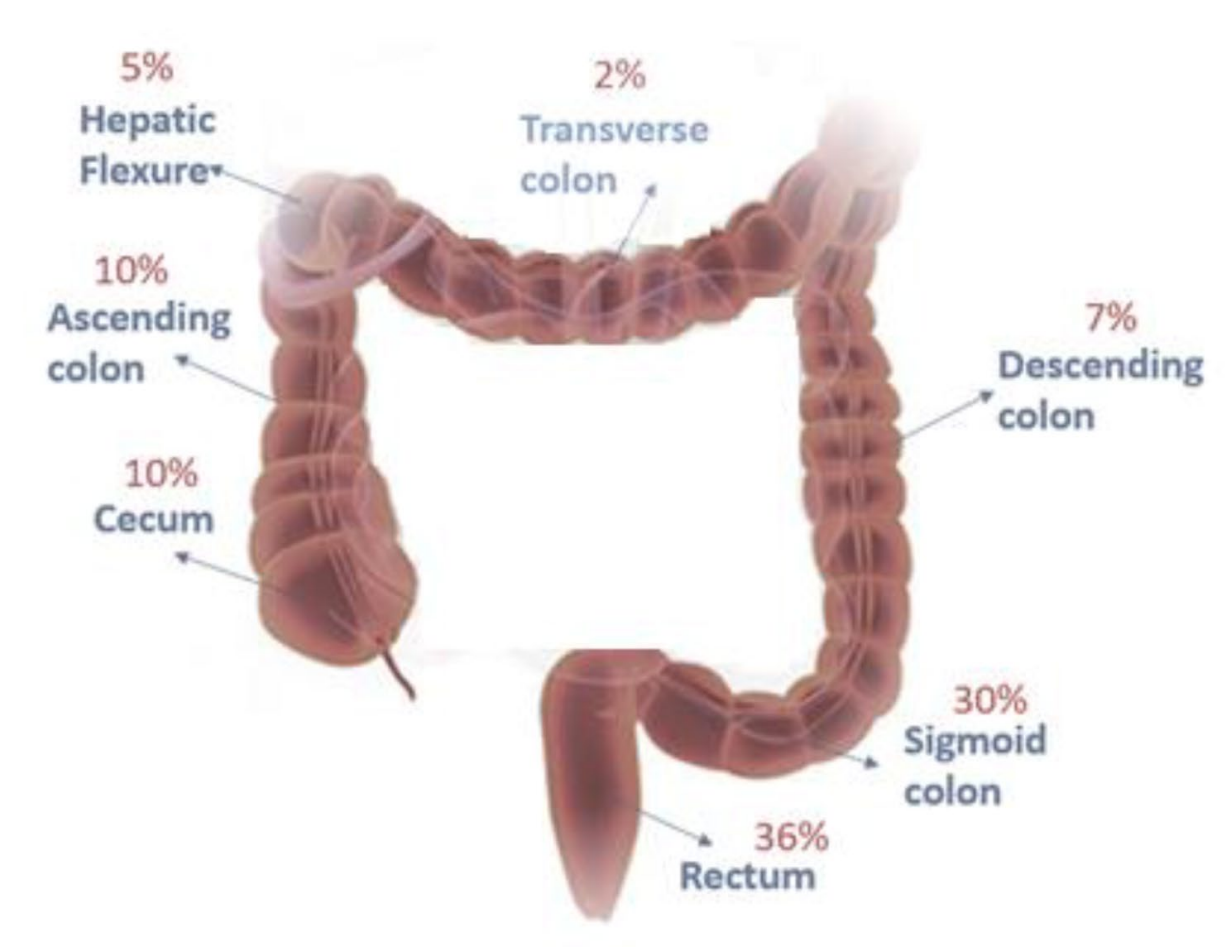
Types of cancer samples examined in this study

According to demographic information, 52% of patients in cancer group were women and 48% of them were men. The age range of the women was between 50 and 60, while men ranged from 50 to 80 years. The individuals in the control group included 45% women and 55% men, with the highest age range between 30 and 40 and 30–50 years for women and men, respectively. The most common symptoms that led to colonoscopy in patients were anemia (34%), abdominal pain (31%), blood in the stool (19%), and rectal bleeding (16%). Figure 1 demonstrates the involvement of different parts of the colon in patients with CRC, obtained after gastroenterology examinations and pathology results. Based on morphological diversity, tissue samples included adenocarcinoma (87%) and adenoma (13%). The tissue samples obtained from the patients are related to the proximal and distal regions of the intestine. Also, Table 2 provides complete descriptions of cancer samples.

**Table 2:** Pathological information of patients with CRC

Patients			Tumor		
Sample ID	Age	Sex	Location	Size	Morphology
C01	42	F	Ascending colon	0.3 × 0.2 × 0.1 cm	Adenocarcinoma
C02	59	M	Hepatic flexure	0.5 × 0.4 × 0.2 cm	Adenocarcinoma
C03	72	M	Rectum	1.5 × 1 × 0.3 cm	Adenocarcinoma
C04	82	F	Sigmoid colon	1.5 × 1 × 0.7 cm	Adenocarcinoma
C05	69	F	Sigmoid colon	0.5 × 0.4 × 0.2 cm	Adenocarcinoma
C06	51	M	Descending colon	0.3 × 0.2 × 0.1 cm	Adenocarcinoma
C07	49	M	Sigmoid colon	0.3 × 0.2 × 0.1 cm	Adenocarcinoma
C08	78	M	Cecum	0.3 × 0.2 × 0.1 cm	Adenocarcinoma
C09	68	M	Cecum	0.5 × 0.3 × 0.2 cm	Adenocarcinoma
C10	48	F	Rectum	0.8 × 0.6 × 0.2 cm	Adenoma
C11	76	F	Cecum	1 × 1 × 0.3 cm	Adenocarcinoma
C12	27	M	Ascending colon	0.6 × 0.4 × 0.2 cm	Adenocarcinoma
C13	51	F	Rectum	1 × 0.7 × 0.3 cm	Adenocarcinoma
C14	84	M	Rectum	0.3 × 0.2 × 0.1 cm	Adenoma
C15	70	F	Rectum	0.3 × 0.2 × 0.1 cm	Adenocarcinoma
C16	76	F	Hepatic flexure	0.3 × 0.2 × 0.1 cm	Adenocarcinoma
C17	56	F	Sigmoid colon	0.5 × 0.3 × 0.2 cm	Adenocarcinoma
C18	65	M	Ascending colon	0.5 × 0.3 × 0.2 cm	Adenocarcinoma
C19	51	M	Sigmoid colon	1 × 0.7 × 0.3 cm	Adenocarcinoma
C20	49	M	Sigmoid colon	0.3 × 0.2 × 0.1 cm	Adenocarcinoma
C21	63	F	Sigmoid colon	1 × 0.8 × 0.2 cm	Adenoma
C22	58	M	Sigmoid colon	0.9 × 0.7 × 0.3 cm	Adenocarcinoma
C23	64	M	Descending colon	0.3 × 0.2 × 0.1 cm	Adenocarcinoma
C24	52	M	Rectum	1 × 0.9 × 0.2 cm	Adenocarcinoma
C25	58	F	Ascending colon	0.6 × 0.2 × 0.2 cm	Adenoma
C26	45	M	Descending colon	0.7 × 0.5 × 0.2 cm	Adenocarcinoma
C27	56	F	Rectum	0.3 × 0.2 × 0.1 cm	Adenocarcinoma
C28	86	M	Rectum	0.3 × 0.2 × 0.1 cm	High grade glandular dysplasia
C29	73	M	Rectum	0.3 × 0.2 × 0.1 cm	Adenocarcinoma
C30	59	F	Rectum	0.3 × 0.2 × 0.1 cm	Adenocarcinoma
C31	63	F	Rectum	0.3 × 0.2 × 0.1 cm	Adenocarcinoma
C32	73	M	Cecum	1 × 0.5 × 0.5 cm	Adenocarcinoma
C33	57	M	Sigmoid colon	0.7 × 0.6 × 0.1 cm	Adenocarcinoma
C34	58	F	Sigmoid colon	0.6 × 0.5 × 0.2 cm	Adenocarcinoma
C35	71	F	Rectum	1.5 × 1 × 0.2 cm	Adenoma
C36	62	M	Transverse colon	0.8 × 0.5 × 0.2 cm	Adenocarcinoma
C37	78	M	Rectum	0.3 × 0.2 × 0.1 cm	Adenocarcinoma
C38	78	F	Sigmoid colon	0.3 × 0.2 × 0.1 cm	Adenocarcinoma
C39	53	F	Sigmoid colon	0.3 × 0.2 × 0.1 cm	Adenocarcinoma
C40	66	F	Rectum	0.3 × 0.2 × 0.1 cm	Adenocarcinoma

F: female, M: male

### The expression level of target genes

**Fig. 2:**
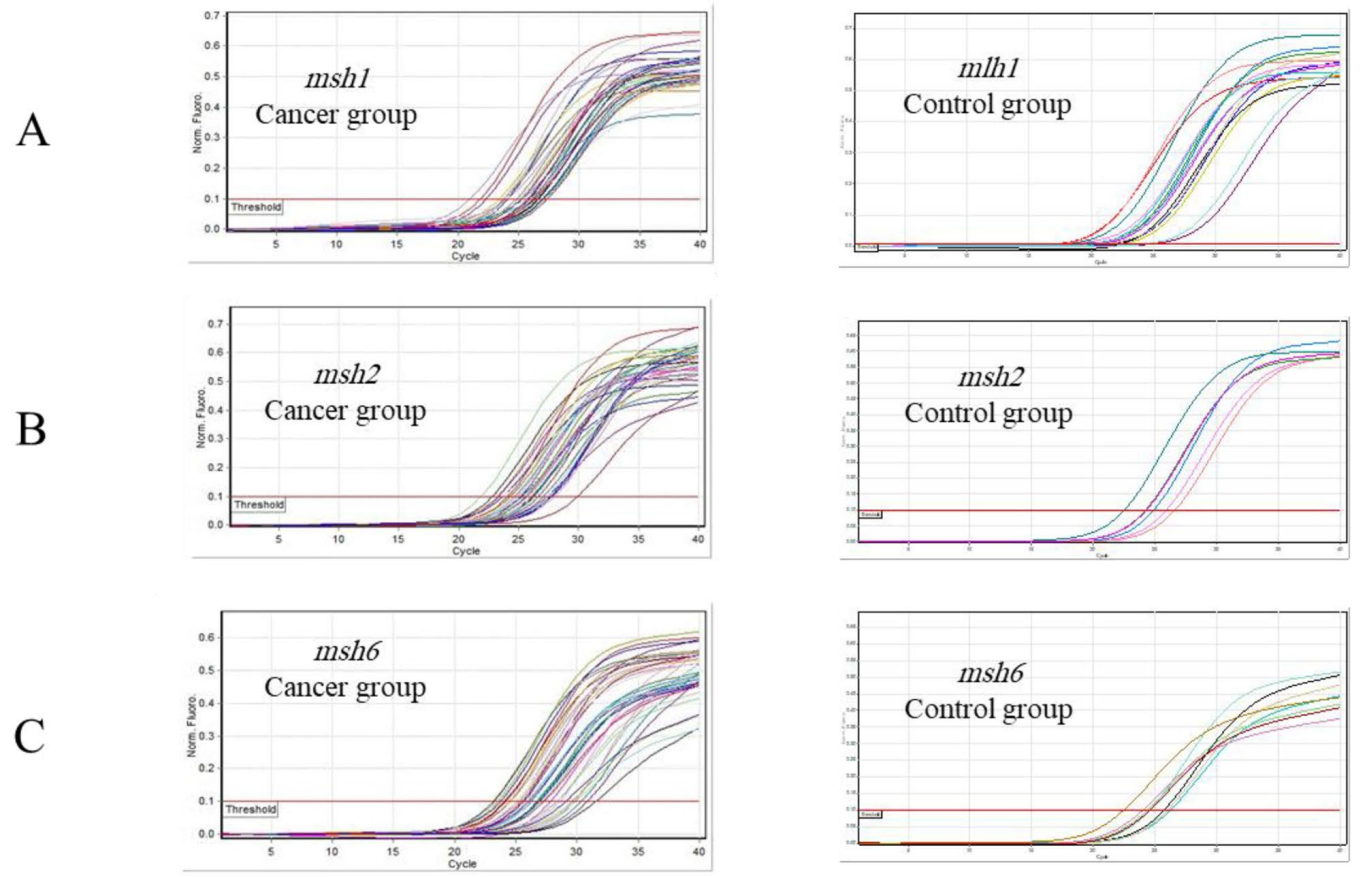
Real-Time PCR progression diagram for (**A**) *mlh1*, (**B**) *msh2*, and (**C**) *msh6* genes in cancer and control groups

**Fig. 3:**
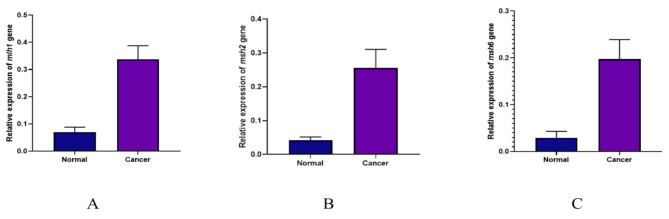
Comparison of the expression level of (**A**) *mlh1*, (**B**) *msh2*, and (**C**) *msh6* genes in cancer and control groups

**Fig. 4:**
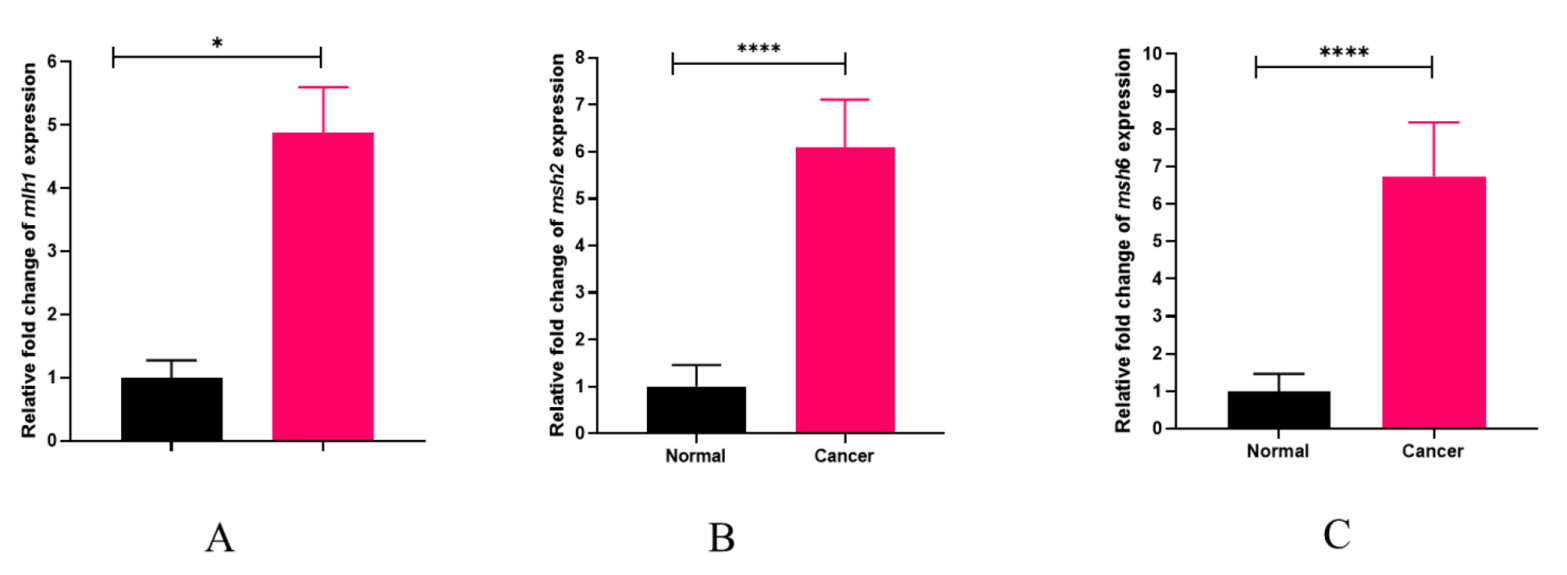
Fold change analysis of *mlh11, msh2, and msh6* genes expression in the cancer group relative to the control groups

**Fig. 5:**
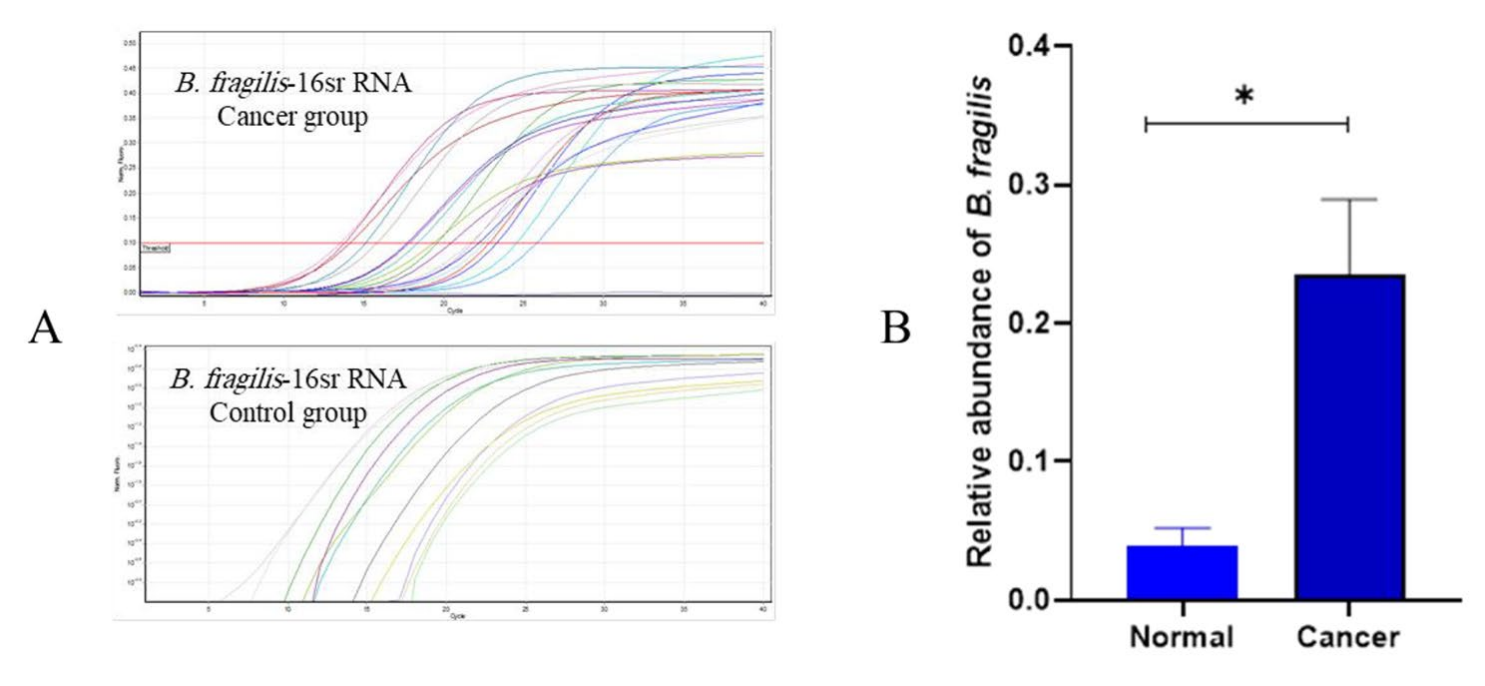
(**A**) Real-Time PCR progression diagram and (**B**) Comparison of the presence of *B. fragilis- 16srRNA* gene in cancer and control groups

The *gapdh* gene was utilized as a control to investigate the expression of *mlh1*, *msh2*, and *msh6*. Real-Time PCR was used in order to estimate *gapdh* gene expression levels in control and cancer groups. Based on obtained results, a comparison of *mlh1* gene expression in control and cancerous groups indicates that the *mlh1* gene in the cancer group significantly increased compared to the control group (*P* value = 0.0139) (Fig. 2). Moreover, *msh2* gene significantly increased in cancer group compared to the control group (*P* value = 0.0128) (Fig. 2). Comparison of *msh6* expression in healthy individuals and cancer patients presented a significant elevation in *msh6* expression in the cancer group compared to the control one (*P* value = 0.0001) (Fig. 2). Figure 3 contains information about the expression of genes in the control and cancer groups. Based on fold change analysis of MMR genes, the level of *mlh1* gene expression was 5 times higher in the cancer group in comparison with the control group (*P* value = 0.0139). Additionally, *msh2* and *msh6* genes expression increased by 6 and 7 times in cancer group compared to the control group, respectively (*P* value = 0.0128 and *P* value = 0.0001) (Fig. 4). The relative abundance of the bacterium was estimated using the *16srRNA* gene primers specific for *B. fragilis*. The results showed that the frequency of *B. fragilis* in the cancer group was significantly higher compared to the control group (*P* value = 0.0378) (Fig. 5).

### *B. fragilis* abundance in cancer and control groups

Real-time PCR was performed using *16s rRNA* specific for *B. fragilis* for all samples. The relative frequency of *B. fragilis* was significantly higher (80%) in the cancer group compared to the control group (50%). The prevalence of *B. fragilis* in men with cancer was higher than in women, so that the presence of this bacterium was reported in 55% in men and 44% in women. The prevalence of *B. fragilis* varied among cancer patients of different genders and age groups, with a higher prevalence observed among men and women aged 50–60.

### Relative abundance of *B. Fragilis* in different types of CRC samples

In another part of the study, the relationship between the location of the tumor and the frequency of *B. fragilis* was investigated, which showed that *B. fragilis* was observed in 68.8% of tumors located in the distal part of the colon and in 31.2% of tumors located in the proximal part of the colon and rectum. Consequently, tumors located in the distal part of the colon were more associated with *B. fragilis*. Results indicated *B. fragilis* was more prevalent in cancer specimens with adenocarcinoma morphology than in other morphologic types. *B. fragilis* was detected in 67.5% of cancer specimens with adenocarcinoma morphology and 4% with adenoma morphology. Results also showed different relative distribution of *B. fragilis* in various parts of the large intestine. Accordingly, the most abundance was observed in the rectum, sigmoid colon, cecum, ascending colon, descending colon, hepatic flexion, and transverse colon, respectively.

### Changes in the relative expression levels of the selected genes in the presence and absence of ***B. fragilis***

**Fig. 6:**
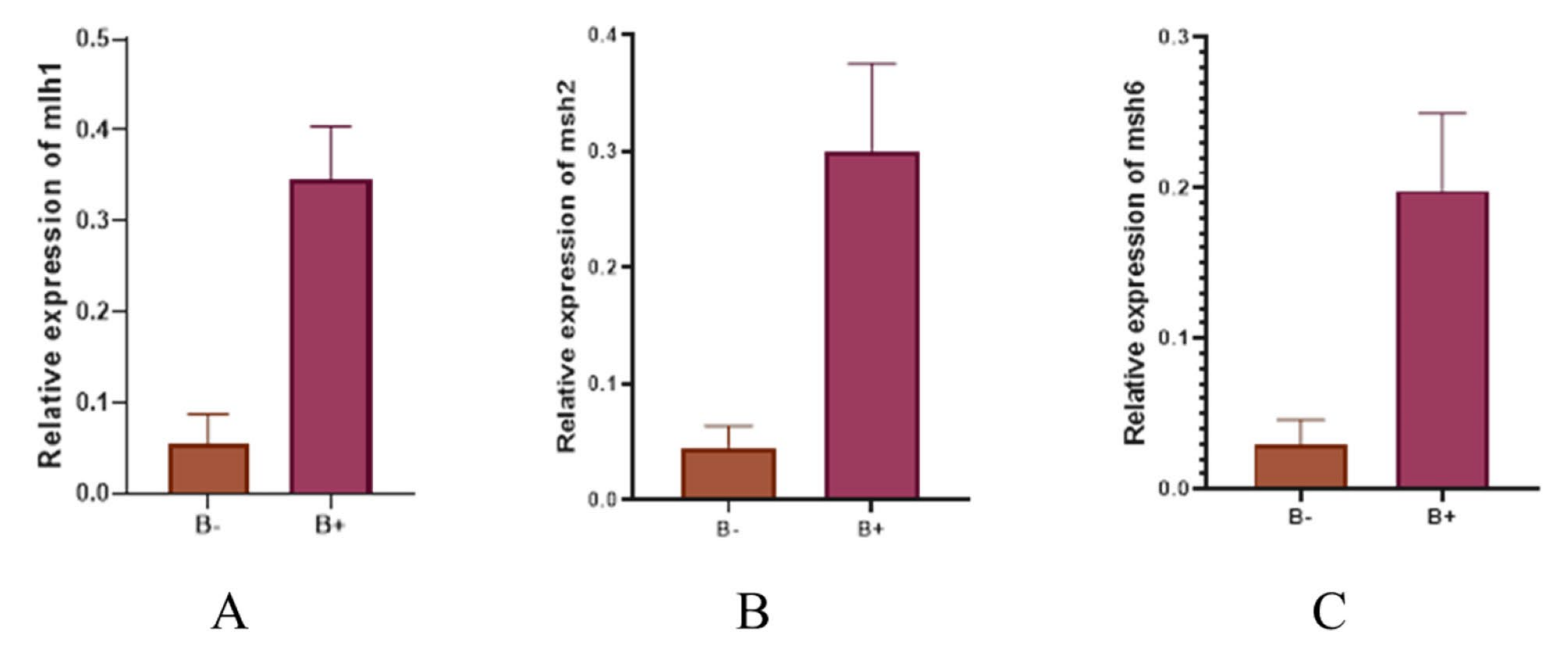
Comparison of the relative presence of *B. fragilis- 16srRNA* and the expression level of (**A**) *mlh1*, (**B**) *msh2*, and (**C**) *msh6* genes in cancer samples

Specifically, the level of expression of *mlh1*, *msh2*, and *msh6* was compared in the cancer group with and without the presence of *B. fragilis. This* indicated an increase in the expression of the mentioned genes in cancerous samples in the presence of *B. fragilis* compared to conditions where *B. fragilis* is not present. In order to evaluate the expression level of *mlh1*, *msh2*, and *msh6* genes, a fold change analysis was done. Based on our results, *msh2* and *msh6* expression levels were raised approximately 6.5 times in the cancer group compared to the control group, and *mlh1* expression levels by about 5 times (Fig. 6).

## Discussion

CRC is one of the most common types of cancer diagnosed worldwide. The occurrence of CRC has been attributed to various factors, with the age of the affected individual being recognized as one of the most significant known risk factors [16]. Several reports indicate that the risk of CRC increases significantly during the fifth decade of life. Despite this, CRCs are rare in individuals under the age of 50 [17]. The age range of patients in this study was 50 to 80 years. A total of 48% of this population was male and 52% was female. In a study conducted by Mirzapoor Abbasabadi et al. in Iran, the age range of the patients was 59.5 years, and 59.6% of the subjects were men, which was different from our study [18]. The results of the current study also demonstrated a higher commonness of CRC in the left part of the colon than in the right part, which was similar to Raza et al., study [19]. In contrast to our study, Komiya et al. found a higher incidence of CRC in the ascending colon. Their results suggest that CRC occurs more frequently in the rectum [20]. Considering the importance of CRC, studying its causes is crucial. It is possible to provide effective prevention and treatment by understanding the causes of these diseases. There are several molecular methods for the early diagnosis of CRC, which can be mentioned as Germ-line APC mutations, mutant alleles of K-*ras* genes, and alteration in MMR genes [21, 22]. The findings of this study show a significant increase in the relative expression of *msh2*, *msh6*, and *mlh1* genes in cancer samples compared to the control group. Many studies have investigated the *mlh1* gene and its role in cancers, especially CRC [23–25]. Defects in MMR genes (*mlh1*, *msh2*, *msh6*) lead to MSI, which is characteristic of hereditary non-polyposis CRC. MSI is a state of genetic variability (prone to mutation) that results from impaired DNA mismatch repair. However, high-frequency MSI occurs in approximately 15% of CRC and other tumors, where MMR defects are caused by epigenetic inactivation of the *mlh1* gene by DNA methylation [26]. A study conducted by Engel et al. in 2019 on tumor tissue stated that the risk of adenoma due to mutation of *msh2* and *msh6* genes is significantly higher compared to *mlh1* [27]. According to the results of the present study, the expression level of *the mlh1* gene in cancer samples increased about 5 times compared to the control group, but this increase was less compared to the other two genes. According to another study conducted by Wang et al. in 2019, the expression level of *msh2* and *mlh1* was examined in the tumor tissue of patients after surgery. In 91% of colorectal carcinomas, the *mlh1* gene was not expressed [28]. Mutations in the *mlh1* and *msh2* genes are primarily responsible for the decrease in expression of these genes. Due to the dominance of these two genes in the MMR system, their detection is imperative to understanding the pathogenesis of sporadic CRC [29, 30]. An additional gene of the MMR system examined in this study is *msh2*, which encodes a protein vital to DNA repair. In a study conducted by Liccardo et al. in Italy in 2020, it was observed that *msh2* gene expression in cancer samples was increased compared to the control group. It has also been mentioned that the overexpression of *mlh1* or *msh2* genes causes apoptosis or mutated and genetically unstable phenotype [31]. According to several studies, it was proven that the overexpression of *mlh1* and *msh2* genes potentially leads to adverse consequences. When these two genes were upregulated in vitro under the control of the cytomegalovirus promoter, apoptosis was induced in a human cell line [31]. In the results of the present study, it was observed that the expression level of *the msh2* gene in cancer samples is almost 6 times higher than in the control group, which is in line with recent studies. In Ekundina et al., study, the mean percentage reactivity of *msh2* in normal, colonic polyps, and colorectal carcinoma was 43.2%, 56.6%, and 90.1% respectively, while the mean percentage reactivity for msh6 was 40.5%, 56.2% and 92% respectively [32]. The level of *msh2* and pms2 protein expression has a positive relationship with tumor size, the degree of tumor invasion to the depth of the tissue, and metastasis to the lymph nodes [33]. According to the studies, mutations related to the expression of the *msh6* gene are associated with a lower risk of cancer compared to the mutations of the *mlh1* or *msh2* genes, and those who carry mutations in the expression of the *msh6* gene at an older age are also more likely to develop CRC.

Several studies have emphasized the significance of the *mlh1* and *msh2* genes in the MMR system. Mutations in either of these genes lead to a loss of function and contribute to tumor formation, particularly in the proximal colon. Hyper methylation, a common occurrence in sporadic tumors, is notably more prevalent than in MSI-positive hereditary tumors. Additionally, multiple studies have indicated that the overexpression of the *mlh1* gene and/or the *msh2* gene is linked to tumor metastasis in various organs.

In addition to changes in MMR gene expression, alterations in the abundance of gut microbiota can also be seen in CRC patients, and identifying these two factors as biomarkers for diagnosis is crucial. The results of examining the expression of *msh2*, *msh6*, and *mlh1* genes in comparison with the presence and absence of *B. fragilis* in cancer samples showed that in cancer samples with *B. fragilis*, there is a greater increase in expression than in cancer samples without this bacterium. *B. fragilis* is regarded as one of the most influential pathogens in the occurrence and spread of colon cancer [34]. According to the results of the present study, the relative frequency of *B. fragilis* in cancer samples has increased about 5 times compared to control samples. Dadgar-Zankbar et al. conducted a study in Iran and found *B. fragilis* was significantly higher in tumor tissues than in adjacent healthy samples (100% vs. 86% respectively) [35]. Several studies have stated that *B. fragilis* toxin is associated with various diseases, including CRC, which can be referred to the study conducted by Boleij et al. in 2015 on intestinal mucosa samples from patients with intestinal neoplasia [36]. These results indicated that CRC is associated with the *B. fragilis* toxin gene in the late stages. They also stated that exposure to *B fragilis* toxin is common, which may be a risk factor for developing CRC. Based on all of these findings, it is pertinent to investigate the abundance of this bacterium in CRC samples. This will enable us to predict cancer progression more quickly and prevent its development. Furthermore, because MMR genes play an important role in cancer development and progression, further studies may be able to establish their importance as factors in the proper diagnosis of cancer or its advanced stage. The limitations of the current study included low sample size, lack of access to samples of different stages of CRC to evaluate biomarkers’ expression in each stage, lack of investigation of fecal microbiota samples for further confirmation and in-depth verification of mechanisms by which *B. fragilis* lead to the damaging effect on the gastrointestinal tract.

## Conclusion

This study reveals heightened expression of specific MMR genes in cancer samples compared to controls. Moreover, CRC biopsy samples exhibit increased bacterial frequency compared to healthy counterparts. Significantly elevated expression of the examined MMR genes is observed in *B. fragilis*-positive cancer samples versus those without this bacterium. Investigating *B. fragilis* presence in confirmed or suspected CRC samples is crucial for expedited cancer diagnosis and prevention. Positive molecular diagnostic tests or elevated risk marker expression potentially identify CRC patients eligible for surveillance or intervention.

## Data Availability

Data are available from the corresponding author upon reasonable request.
